# *In Situ* Gene Expression Responsible for Sulfide Oxidation and CO2 Fixation of an Uncultured Large Sausage-Shaped *Aquificae* Bacterium in a Sulfidic Hot Spring

**DOI:** 10.1264/jsme2.ME16013

**Published:** 2016-06-07

**Authors:** Satoshi Tamazawa, Kyosuke Yamamoto, Kazuto Takasaki, Yasuo Mitani, Satoshi Hanada, Yoichi Kamagata, Hideyuki Tamaki

**Affiliations:** 1 Bioproduction Research Institute, National Institute of Advanced Industrial Science and Technology (AIST)Central 6, Higashi 1–1–1, Tsukuba, Ibaraki 305–8566Japan; 2 Graduate School of Life and Environmental Sciences, University of Tsukuba1–1–1 Ten-noudai, Tsukuba, Ibaraki 305–8572Japan

**Keywords:** sulfur-turf microbial mats, uncultured *Aquificae* bacterium, *in situ* gene expression, sulfide oxidation, primary production

## Abstract

We investigated the *in situ* gene expression profile of sulfur-turf microbial mats dominated by an uncultured large sausage-shaped *Aquificae* bacterium, a key metabolic player in sulfur-turfs in sulfidic hot springs. A reverse transcription-PCR analysis revealed that the genes responsible for sulfide, sulfite, and thiosulfate oxidation and carbon fixation via the reductive TCA cycle were continuously expressed in sulfur-turf mats taken at different sampling points, seasons, and years. These results suggest that the uncultured large sausage-shaped bacterium has the ability to grow chemolithoautotrophically and plays key roles as a primary producer in the sulfidic hot spring ecosystem *in situ*.

Chemolithoautotrophic *Aquificae* bacteria are widely distributed and often dominate in various sulfidic geothermal environments such as terrestrial hot springs (5, 11, 27, 29, 35, 36) and deep-sea hydrothermal vents (20, 30, 34). Due to their abundance in sulfidic environments, these *Aquificae* bacteria are considered to be involved in the sulfur cycle and primary production in these environments.

The white microbial mat, the so-called “sulfur-turf” found in streams of sulfidic, circumneutral, and hypoxic hot spring waters worldwide (13, 17, 33, 40), is an assemblage of white filaments mainly composed of uncultured *Aquificae* bacteria phylogenetically related to the genus *Sulfurihydrogenibium*, and elemental sulfur particles or aragonite (13, 17). The dominant organism, often called a large sausage-shaped bacterium (LSSB) because of its conspicuous size (a cell length of 5–40 μm) and shape, has not yet been cultivated. Since the uncultured LSSB thrives in sulfidic (~0.1 mM) and organic-poor (<0.4 mg L^−1^ of total organic carbon) hot spring water streams and a large amount of elemental sulfur particles are precipitated around the cells (14, 15, 23, 26), sulfide is likely to be the main energy source for growth. However, the physiological features of the uncultured LSSB remain unclear because of its unculturability, which hampers all culturedependent analyses.

In order to clarify the metabolic functions of the uncultured LSSB, we recently conducted a draft genome analysis using the metagenomic library of a sulfur-turf collected from Nakabusa hot spring in Japan (39). The genomic data obtained showed that the uncultured LSSB possesses key genes associated with sulfide, sulfite, and thiosulfate oxidation, microaerobic respiration, and carbon fixation via the reductive TCA (rTCA) cycle, indicating the potential for chemolithoautotrophic growth. However, there has been no evidence to show that these predicted genes of the uncultured LSSB are indeed active *in situ*. In order to verify this, the present study investigated the *in situ* expression profiles of the genes associated with inorganic sulfur oxidation and carbon fixation of the uncultured LSSB in the sulfur-turf by using a reverse transcription (RT)-PCR approach based on the metagenomic data we obtained from the environment.

Sulfur-turf samples were collected from a sulfidic hot spring in Nakabusa, Japan (36°23.482N, 137°44.883E) in November 2011 and in July and November 2013. The sulfur-turfs developed on a concrete wall down to 70 cm from the discharge point of hot spring water. The temperature and pH of the stream at sampling points were measured using CT-280WR (CUSTOM, Tokyo, Japan) and a B-211 COMPACT pH METER (HORIBA, Kyoto, Japan), respectively. Samples were collected at three points within the same sulfur-turf using sterilized tweezers (Fig. 1). The collected samples were immediately washed once with 10 mM KH_2_PO_4_/K_2_HPO_4_ (pH 8.0) buffer and then immersed in RNAlater solution (Ambion, TX, USA). The samples were transported to our laboratory on ice within 8 h and stored in the laboratory at −20°C until used. Each sample was transferred to a Lysing Matrix E tube (MO-BIO, CA, USA), suspended with RLT buffer contained in the RNeasy Mini Kit (Qiagen, Hilden, Germany), and then disrupted by bead-beating using FastPrep FP120 (BIO 101 SAVANT, NY, USA). The suspension was centrifuged at 13,000×*g* at 4°C for 10 min and the supernatant was mixed with a 0.55 volume of ethanol. The mixture was transferred into an RNeasy mini spin column and subjected to further purification steps using an RNeasy Mini Kit according to the manufacturer’s instructions. A DNase treatment was performed using an RNase-free DNase Set (Qiagen) and TURBO DNase (Ambion) to further remove trace genomic DNA contamination. The reaction product was purified with phenol/chloroform (1:1 [v/v]) and then washed once with chloroform. Total RNA was precipitated with ethanol, washed with 70% (v/v) ethanol, and then resuspended in RNase-free water. Purified total RNA was analyzed by 0.8% (w/v) agarose gel electrophoresis in order to check for RNA degradation. RNA concentrations were quantified using a Qubit fluorometer (Invitrogen, CA, USA) and Quant-iT RNA Assay Kit (Invitrogen) according to the manufacturer’s instructions.

In our previous draft genome analysis of the uncultured LSSB, 1,472 ORFs were predicted (39). In order to obtain further insights into its metabolic potential, functional annotations were performed using the KEGG Automatic Annotation Server (KAAS) Ver. 2.0 (21) and Transporter Automatic Annotation Pipeline (TransAAP) (28). The genomic features revealed in this analysis did not positively support the possibility that the uncultured LSSB grows heterotrophically for the following reasons: (i) any gene for a primary enzyme in the glycolysis pathway (*i.e.* pyruvate kinase) was not found, although all genes associated with glycogenesis and the TCA cycle were identified (Fig. S1). (ii) Any transporters of sugars/oligosaccharides were not annotated by TransAAP (Table S1). (iii) The lack of genes coding pyruvate kinase and the transporters of sugars/oligosaccharides is not likely due to the incompleteness of the draft genome (90% completeness) of the uncultured LSSB because the complete genome of the chemolithoautotrophic sulfur oxidizer *Sulfurihydrogenibium azorense*, which is phylogenetically closely related to the uncultured LSSB, also lacks the genes for pyruvate kinase and transporters of sugars/oligosaccharides, and this strain is, indeed, unable to grow using any carbohydrates. Considering the *in situ* environmental conditions (*i.e.*, the small amount of organic carbon in the discharged hot spring water) and rapid growth rate (doubling time of 80–92 min) of sulfur-turf reported previously (15, 23), it is difficult to infer that the uncultured LSSB grows heterotrophically *in situ*. Therefore, we focused on the *in situ* autotrophic growth activity of the uncultured LSSB, and attempted to verify whether the genes associated with inorganic sulfur oxidation and carbon fixation of the uncultured LSSB are expressed in the sulfur-turf *in situ*.

Based on the metagenomic data of the sulfur-turf collected at the same stream in our previous study (39), the targeted genes of the uncultured LSSB for the RT-PCR analysis were selected and specific primers for these genes were designed using Primer3 (16) as follows: sulfide oxidation-related genes (*dhsU1* and *dhsU2*, sulfide dehydrogenase; *sqrX* and *sqrF*, sulfide-quinone reductase), a sulfite oxidation-related gene (*sorA*, sulfite dehydrogenase), thiosulfate oxidation-related genes (*soxX*, *soxY*, *soxZ*, *soxA*, and *soxB*, the sulfur oxidation (sox) system without *soxC* and *soxD*), a respiration-related gene (*ccoN*, *cbb*-3 type cytochrome *c* oxidase as a terminal oxidase working under microaerobic conditions), the genes responsible for carbon fixation via the reductive TCA cycle (*aclA*, ATP citrate lyase; *fumA*, fumarate hydratase; *forA*, 2-oxoglutarate ferredoxin oxidoreductase), and the gene encoding the RNA polymerase omega subunit (*rpoZ*) as a positive control (Table S2) (39). In addition to these genes, three genes encoding thiosulfate sulfurtransferase (*rhd1*, *rhd2*, and *rhd3*) were newly annotated in the draft genome in this study (GenBank accession numbers LC021536–LC021538, Table S2) and targeted for the RT-PCR analysis. In order to evaluate the specificity of the designed primers, PCR amplification using DNA extracted from a sulfur-turf as a template with TaKaRa Ex Taq Hot Start Version (TaKaRa, Otsu, Japan) was performed as follows: an initial denaturation step at 95°C for 2 min, followed by 30 cycles of denaturation at 95°C for 30 s, annealing at 56°C for 30 s, and a final extension step at 72°C for 5 min. The PCR product was analyzed by 3% (w/v) agarose gel electrophoresis. The remaining PCR product was purified, cloned, and sequenced, as described previously (39).

Two-step RT-PCR was performed using ReverTra Ace-α-(TOYOBO, Tokyo, Japan) with 0.18–0.25 μg of extracted total RNA and a random primer for RT, according to the manufacturer’s instructions. Following PCR using the gene-specific primers (Table S2), electrophoresis and sequencing of the PCR product were performed according to the above procedure.

The temperature and pH of the stream at three sampling points (5, 40, and 68 cm below the discharge point) were 54.2 to 52.2°C and 7.9 to 8.0, respectively. Using the sulfur-turf samples taken at each of the three points in the hot springs, we investigated *in situ* transcription profiles using a RT-PCR analysis. We designed gene-specific primers for these genes and confirmed their specificities by PCR amplification and sequencing. As a result, a single PCR band was successfully obtained from sulfur-turf DNA with the corresponding primer sets designed, and the sequences of the respective PCR products were identical to the target sequences in the draft genome of the uncultured LSSB (data not shown).

Using the designed primer sets, a RT-PCR analysis was performed on total RNA extracted from the three points of the sulfur-turf. The expression of all the genes tested was detected at all three positions of the sulfur-turf (Fig. 1). The amplification of negative controls, RT samples without reverse transcriptase, was not detected (data not shown). The sequences (three to eight clones) of each of the RT-PCR products were identical to the corresponding gene sequence in the uncultured LSSB draft genome (except for *fumA*, 1 mismatch found in 83 nt) (Fig. S2), indicating that the origin of the detected mRNA was the uncultured LSSB. These results suggest that the uncultured LSSB is able to acquire energy using reduced sulfur compounds as electron donors and oxygen as an electron acceptor in the sulfur-turf. Sulfide oxidation by the uncultured LSSB is strongly supported by a previous study demonstrating that sulfide was oxidized to elemental sulfur within a sulfur-turf dominated by the uncultured LSSB (18), while the oxidation of sulfite and thiosulfate by the uncultured LSSB has not yet been demonstrated and needs to be addressed by further physiological and biochemical characterizations. Furthermore, the uncultured LSSB may be capable of fixing CO_2_ through the rTCA cycle in the sulfur-turf. The expression of the genes related to thioautotrophic growth was observed not only at all three positions within the large sulfur-turf, but also in other sulfur-turf samples harvested in different seasons and years (in July and November 2013, data not shown). Considering this stable expression pattern and the abundance of the uncultured LSSB in the sulfur-turf (>90% of the whole bacterial community) (5, 39), it is likely that the uncultured LSSB plays important roles as a sulfur oxidizer and primary producer *in situ*.

Based on the present *in situ* gene expression profile and previous genomic and physiological studies on *Aquificae* and other sulfur-oxidizing bacteria, we hypothesized the active sulfur-oxidation pathways of the uncultured LSSB in the microaerobic and sulfidic environments as follows (Fig. 2): the uncultured LSSB oxidizes sulfide continuously supplied from hot spring water to elemental sulfur by DhsU and Sqr, which have already been characterized biochemically as sulfide-oxidizing enzymes (19, 24, 39). The uncultured LSSB is unlikely to oxidize elemental sulfur in the environment because the abundant precipitation of elemental sulfur was observed around uncultured LSSB cells (39, 40). To date, there has been no evidence for the presence of genes responsible for oxidizing elemental sulfur not only in the draft genome of the uncultured LSSB (39), but also in the complete genomes of *Sulfurihydrogenibium* isolates (31), which also supports the possibility that the uncultured LSSB is unable to oxidize elemental sulfur in the environment. Since sulfide is constantly supplied from hot spring water, sulfide oxidation to elemental sulfur is considered to be the main sulfur oxidation pathway of the uncultured LSSB. On the other hand, sulfide is abiotically oxidized to thiosulfate, elemental sulfur, and sulfite in the presence of oxygen (3, 4), and the expression of genes involving thiosulfate oxidation was detected. Taken together, in addition to sulfide oxidation, the uncultured LSSB may also utilize thiosulfate as one of the energetic substrates. Two pathways have been proposed for thiosulfate utilization: oxidation to sulfate by an incomplete Sox system (7, 32) and disproportionation into sulfur in the form of sulfane sulfur and sulfite by Rhd (2, 8, 9) (Fig. 2). Thiosulfate oxidation to sulfate mediated by the Sox system likely occurs *in situ* because all the genes encoding the Sox system were found in the uncultured LSSB genome and expressed in the sulfur-turf. However, sulfite oxidation to sulfate by Sor currently remains unclear because a gene encoding SorA, which is a part of the SorAB two-subunit system, was found in the draft genome, whereas a gene encoding SorB was not annotated. Further biochemical and physiological studies are needed in order to elucidate the sulfite-oxidizing pathway of the uncultured LSSB in more detail. Nevertheless, the potential ability to utilize various reduced sulfur compounds as energetic substrates may provide the uncultured LSSB with an advantage to predominate in the sulfidic environment.

Recent metagenomic analyses showed the potential of *Aquificae* bacteria dominating in microbial mats or streamers to grow autotrophically by inorganic sulfur oxidation (13, 36). In recent years, Hamamura *et al.* also reported that the expression of some genes involved in thioautotrophic growth under low oxygen conditions was detected in *Sulfurihydrogenibium*-dominated microbial communities from geothermal springs in Yellowstone National Park in the United States (10). The analogies found in the geographically separated fields in Yellowstone National Park (USA) and Nakabusa hot spring field (Japan) imply that the dominance of the thioautotrophic lifestyle is a common feature of the microbial mat in the geothermal sulfidic ecosystem worldwide.

A number of strains within the phylum *Aquificae* have been isolated from various sulfidic environments, and reduced sulfur compound-dependent autotrophic growth is observed in some *Aquificae* isolates (1, 6, 12, 22, 25, 37, 38). The present study clearly demonstrated the *in situ* expression of the key genes involved in reduced sulfur compound oxidation and carbon fixation in the uncultured LSSB belonging to the phylum *Aquificae* by a transcriptional analysis. Our results provide insights into the ecophysiological roles of *Aquificae* bacteria dominating in various sulfidic environments.

## Supplementary Material



## Figures and Tables

**Fig. 1:**
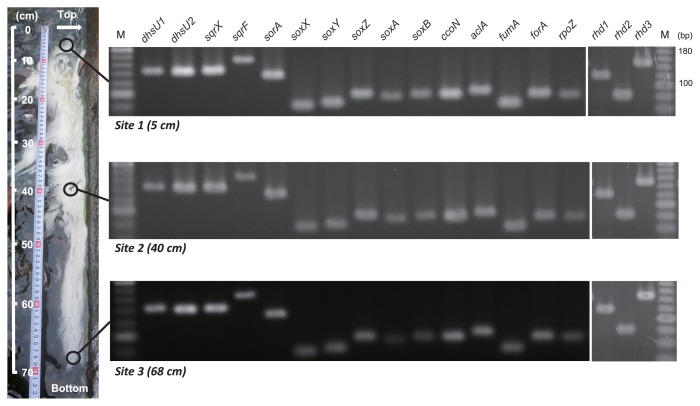
Photos of the sulfur-turf (white microbial mat) thriving in the hot spring stream and RT-PCR detection of transcripts of genes related to reduced sulfur compound oxidation, respiration, and carbon fixation. The white arrow shows the discharge point of the spring. Hot spring water flows downward from the top to the bottom in this figure. *dhsU1* and *dhsU2*, sulfide dehydrogenase; *sqrX* and *sqrF*, sulfide-quinone reductase; *sorA*, sulfite dehydrogenase; *soxX*, *soxY*, *soxZ*, *soxA* and *soxB*, sox complex; *rhd1*, *rhd2* and *rhd3*, thiosulfate sulfurtransferase; *ccoN*, *cbb*-3 type cytochrome *c* oxidase; *aclA*, ATP citrate lyase; *fumA*, fumarate hydratase; *forA*, 2-oxoglutarate ferredoxin oxidoreductase; *rpoZ*, RNA polymerase; M, DNA marker.

**Fig. 2:**
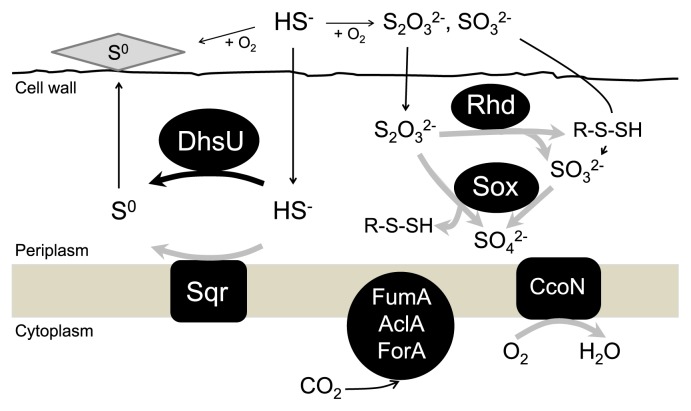
Hypothesized model of the active autotrophic sulfur-oxidation pathway in the uncultured LSSB in the *in situ* sulfur-turf ecosystem. The bold black arrow indicates the reaction that has been biochemically demonstrated in the uncultured LSSB and verified at the transcriptional level in this study. The bold gray arrows show the putative reactions verified at the transcriptional level in this study, but lacking direct biochemical evidence in the uncultured LSSB. The thin black arrows represent the flow of the substrates and products postulated. DhsU, sulfide dehydrogenase; Sqr, sulfide-quinone reductase; Sox, sox complex; Rhd, thiosulfate sulfurtransferase; CcoN, *cbb*-3 type cytochrome c oxidase; AclA, ATP citrate lyase; FumA, fumarate hydratase; ForA, 2-oxoglutarate ferredoxin oxidoreductase.
