# Supplementary material for: In Situ Gene Expression Responsible for Sulfide Oxidation and CO2 Fixation of an Uncultured Large Sausage-Shaped Aquificae Bacterium in a Sulfidic Hot Spring
Source: Microbes Environ. 2016 Jun 7;31(2):194–8. doi: 10.1264/jsme2.ME16013 (PMC4912159; doi:10.1264/jsme2.ME16013)
Supplement: Supplementary file 1 [file 31_194_s1.pdf]

```
LSSB_dhsU1       1 CACACCAAAAGGCTGGAGATTTAGTGTTAAAGTTTGGACTTGCTGATGACAAAGGTTGGGCTCAAATAAATCCAAAAACTTTTGAATCTAAAAATGTAAAAGGCATATATGTTATAGGAGATTCATGTGCAACACCTATGCCAA 144
RT-PCR_dhsU1     1 CACACCAAAAGGCTGGAGATTTAGTGTTAAAGTTTGGACTTGCTGATGACAAAGGTTGGGCTCAAATAAATCCAAAAACTTTTGAATCTAAAAATGTAAAAGGCATATATGTTATAGGAGATTCATGTGCAACACCTATGCCAA 144
                   ************************************************************************************************************************************************

LSSB_dhsU2       1 GTGTATCGGCAGCAAGACAACTTAGAAAATTAAATCCGGATTTATCTATAACTATAATAGACAAAAATCCATTTTTTGTTTCCTGTCCTATGAGTAATCTATACTTGGGAGATTTTTACGAATTTGGCAACCTTTGTTT 139
RT-PCR_dhsU2     1 GTGTATCGGCAGCAAGACAACTTAGAAAATTAAATCCGGATTTATCTATAACTATAATAGACAAAAATCCATTTTTTGTTTCCTGTCCTATGAGTAATCTATACTTGGGAGATTTTTACGAATTTGGCAACCTTTGTTT 139
                   *******************************************************************************************************************************************

LSSB_sqrX        1 TACCACCATTTGCAGGACAACCTATAAAATGGATAGACAAAGATGGAAATGATATTAAAGATAAAGTTTGCAATCCTGCAGGGTTTGTAAAAGTTGATGCTGTTTATGGAAAACCTTATGAAGAACTTGATGGTCCAGATTGGC 144
RT-PCR_sqrX      1 TACCACCATTTGCAGGACAACCTATAAAATGGATAGACAAAGATGGAAATGATATTAAAGATAAAGTTTGCAATCCTGCAGGGTTTGTAAAAGTTGATGCTGTTTATGGAAAACCTTATGAAGAACTTGATGGTCCAGATTGGC 144
                   ************************************************************************************************************************************************

LSSB_sqrF        1 TTGATAGTTGCGTTGGGACAGTCAAAATTAAAGCATAAGGGTATTGAAAACACATACTCTATATGTTCAAATCCGGATGAAACCTTAAAGTATTCAGAAAAATTGGATGAGATAATAAAAAAAGGAAAAGGAAAACTTGCATTTGGTTTTGGTGGTAATCCAAAAGCC 168
RT-PCR_sqrF      1 TTGATAGTTGCGTTGGGACAGTCAAAATTAAAGCATAAGGGTATTGAAAACACATACTCTATATGTTCAAATCCGGATGAAACCTTAAAGTATTCAGAAAAATTGGATGAGATAATAAAAAAAGGAAAAGGAAAACTTGCATTTGGTTTTGGTGGTAATCCAAAAGCC 168
                   ********************************************************************************************************************************************************************

LSSB_sorA        1 AACCTCTCCCAGCGGATAACGGCTATCCATTGAGACTTATTGTGCCTAAGTTATACTCTTGGAAAGTGCAAAATTTGTTAAGGGTATAGAGTTTATTCCAGAAGACAGACCAGGGTTTTGGGAACAAAGAGGG 134
RT-PCR_sorA     41 AACCTCTCCCAGCGGATAACGGCTATCCATTGAGACTTATTGTGCCTAAGTTATACTCTTGGAAAGTGCAAAATTTGTTAAGGGTATAGAGTTTATTCCAGAAGACAGACCAGGGTTTTGGGAACAAAGAGGG 134
                   **************************************************************************************************************************************

LSSB_soxX        1 GGGATAGAAAGTCCGGAAGGTAAAAGTGTTATGTTGAAAGATGTTCCACCTGAACCAAGACTTTATGCCGTTCCTCCTGA 80
RT-PCR_soxX      1 GGGATAGAAAGTCCGGAAGGTAAAAGTGTTATGTTGAAAGATGTTCCACCTGAACCAAGACTTTATGCCGTTCCTCCTGA 80
                   ********************************************************************************

LSSB_soxY        1 GTATCTGCAGCCGAATTGGTTGATAATCCTCCACCAAAGAAACCTTTTGATGAAGCATTAAAAGAGATAACGGGTGGAAAGGCT 84
RT-PCR_soxY      1 GTATCTGCAGCCGAATTGGTTGATAATCCTCCACCAAAGAAACCTTTTGATGAAGCATTAAAAGAGATAACGGGTGGAAAGGCT 84
                   ************************************************************************************

LSSB_soxZ        1 AAGGACAAAGAAACGGGAAAATACATAGCAGCCCATTACATTACAACTGTTGAAGTTTACTATGGAGACGAAAAAATTACATGGATGGATTTATCCGG 98
RT-PCR_soxZ      1 AAGGACAAAGAAACGGGAAAATACATAGCAGCCCATTACATTACAACTGTTGAAGTTTACTATGGAGACGAAAAAATTACATGGATGGATTTATCCGG 98
                   **************************************************************************************************

LSSB_soxA        1 ATCCTGGGGAAGTTTTTGCTCAAGAAGTTGGTGGAGCATTGTTTAACAAACCTATGGGAAGCTCAAACAAATCATGTGCTTCTTGCCACAGTGA 94
RT-PCR_soxA      1 ATCCTGGGGAAGTTTTTGCTCAAGAAGTTGGTGGAGCATTGTTTAACAAACCTATGGGAAGCTCAAACAAATCATGTGCTTCTTGCCACAGTGA 94
                   **********************************************************************************************

LSSB_soxB        1 ACGGCATTAAGCCAAATACGGTAAGAGCTTATTTTGATACTTACATAGGATTTGAAGAACTTGCAAAAAAAATATGGAAAGATGGGTGGTGTTGCCTA 97
RT-PCR_soxB      1 ACGGCATTAAGCCAAATACGGTAAGAGCTTATTTTGATACTTACATAGGATTTGAAGAACTTGCAAAAAAAATATGGAAAGATGGGTGGTGTTGCCTA 97
                   *************************************************************************************************

LSSB_ccoN        1 ATCGGCTTACAAGCAGCAGTATTGATAACGGTTGTTGCAATACTTTTCTTAGGTCAAGCTGACAACGTTGAATGGGGAGAAACTCCTTGGTGGATCGA 98
RT-PCR_ccoN      1 ATCGGCTTACAAGCAGCAGTATTGATAACGGTTGTTGCAATACTTTTCTTAGGTCAAGCTGACAACGTTGAATGGGGAGAAACTCCTTGGTGGATCGA 98
                   **************************************************************************************************

LSSB_aclA        1 AACTTGGGCATCTGGATTTGTAGATATGGTTATAAAAGTAGTGGCGGACCATGGACCTGCCGTTGCTGGAGCCCATAACACAAAAGTTACCGCAAGAGCTGGAA 104
RT-PCR_aclA      1 AACTTGGGCATCTGGATTTGTAGATATGGTTATAAAAGTAGTGGCGGACCATGGACCTGCCGTTGCTGGAGCCCATAACACAAAAGTTACCGCAAGAGCTGGAA 104
                   ********************************************************************************************************

LSSB_fumA        1 CCTTGGATTTGGAGGAACAACGACTGCGGTAGATGTAAAAATAGAGATAGCACCTTGCCATATAGCATCTTTACCAGTTGCCG 83
RT-PCR_fumA      1 CCTTGGATTTGGAGGAACAACAACTGCGGTAGATGTAAAAATAGAGATAGCACCTTGCCATATAGCATCTTTACCAGTTGCCG 83
                   ********************* *************************************************************

LSSB_forA        1 TGTTACATGCGGAAAACCAACAGGATGTTTTTGATTTTACCCTTGCAGCTTTTGCCATAGCTGAAATGGTAGATGTATACATACCTGTTGCGGTAGCA 98
RT-PCR_forA      1 TGTTACATGCGGAAAACCAACAGGATGTTTTTGATTTTACCCTTGCAGCTTTTGCCATAGCTGAAATGGTAGATGTATACATACCTGTTGCGGTAGCA 98
                   **************************************************************************************************

LSSB_rpoZ        1 ACGAAACTGGTGCTGAGACATATGTAACAGAAGAAGGAATCCCACTAAAAAAGACCGTTATTGCAATAGATGAGATAGCAACCGGGAAAGCAAAA 95
RT-PCR_rpoZ      1 ACGAAACTGGTGCTGAGACATATGTAACAGAAGAAGGAATCCCACTAAAAAAGACCGTTATTGCAATAGATGAGATAGCAACCGGGAAAGCAAAA 95
                   ***********************************************************************************************

LSSB_rhd1        1 AAAGAAAAAGAGGCGATGCTCGTCCCACTTATGAGTTTACCAAGAGTAATTAACAGTCTTCCAAAAGATAAAGATATATATGTATTTTGTAGAAGTGGAAATAGAAGCCTTCAAGCAA 118
RT-PCR_rhd1      1 AAAGAAAAAGAGGCGATGCTCGTCCCACTTATGAGTTTACCAAGAGTAATTAACAGTCTTCCAAAAGATAAAGATATATATGTATTTTGTAGAAGTGGAAATAGAAGCCTTCAAGCAA 118
                   **********************************************************************************************************************

LSSB_rhd2        1 TGCATTGGCTTTACAGGATGTAGCTTTAAAAAATAATGAAAAACAACGTTGTAAATACCTTCCTCTATGTCCCCAAACAGC 81
RT-PCR_rhd2      1 TGCATTGGCTTTACAGGATGTAGCTTTAAAAAATAATGAAAAACAACGTTGTAAATACCTTCCTCTATGTCCCCAAACAGC 81
                   *********************************************************************************

LSSB_rhd3        1 CACCCCAAGAATACGAAAAAGATGGTCATATCCCAAATTCTATTTTAATTCCAGTTCAAATTTTACCTCAGTATATTAAGGAGTTAGAAAAGTTTAAAGATAAAAAAGTGTTAGTATACTGTCGATCTGGAAACAGAAGTGTTCTGGC 148
RT-PCR_rhd3      1 CACCCCAAGAATACGAAAAAGATGGTCATATCCCAAATTCTATTTTAATTCCAGTTCAAATTTTACCTCAGTATATTAAGGAGTTAGAAAAGTTTAAAGATAAAAAAGTGTTAGTATACTGTCGATCTGGAAACAGAAGTGTTCTGGC 148
                   ****************************************************************************************************************************************************
```

Figure S1. Sequence alignments between the uncultured LSSB draft genome and RT-PCR products.

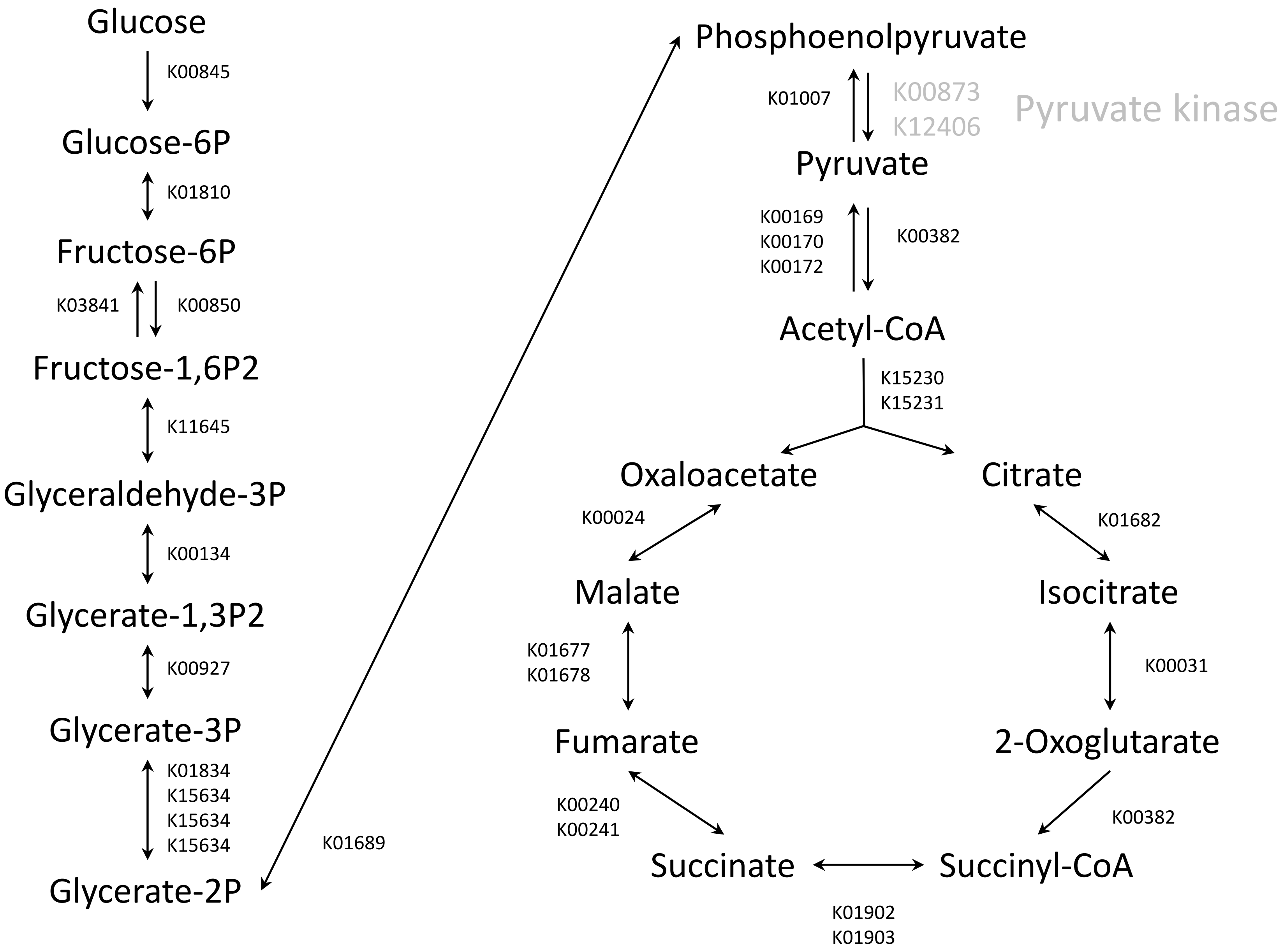

Figure S2. KEGG pathways of glycogenesis and TCA cycle in the uncultured LSSB. Black and gray KEGG numbers show annotated- and not annotated-genes in the draft genome of the uncultured LSSB, respectively. No genes (K00873 and K12406) encoding the key enzyme, pyruvate kinase, were annotated in the graft genome.

Table S1. Transporter proteins in the draft genome of the uncultured LSSB.

Locus tag	Family ID	Family TC	Substrate
LC145186	Oxa1	2.A.9	60 KD inner membrane protein OxaA homolog
LC145153	APC	2.A.3	amino acid
LC145216	Amt	1.A.11	ammonium
LC145150	ArsB	2.A.45	arsenite (ArsB)
LC145171	P-ATPase	3.A.3	calcium ion/manganese ion
LC145123	CDF	2.A.4	cation efflux
LC145162	CDF	2.A.4	cation efflux
LC145199	CDF	2.A.4	cation efflux
LC145184	ABC	3.A.1	cobalt
LC145148	RND	2.A.6	cobalt/zinc/cadmium ion efflux (HME subfamily)
LC145178	RND	2.A.6	cobalt/zinc/cadmium ion efflux (HME subfamily)
LC145180	RND	2.A.6	cobalt/zinc/cadmium ion efflux (HME subfamily)
LC145206	RND	2.A.6	cobalt/zinc/cadmium ion efflux (HME subfamily)
LC145209	RND	2.A.6	cobalt/zinc/cadmium ion efflux (HME subfamily)
LC145215	RND	2.A.6	cobalt/zinc/cadmium ion efflux (HME subfamily)
LC145108	P-ATPase	3.A.3	copper ion
LC145205	MerTP	9.A.2	copper ion
LC145132	ABC	3.A.1	D-methionine
LC145181	ABC	3.A.1	daunorubicin
LC145200	ABC	3.A.1	daunorubicin
LC145117	ABC	3.A.1	dipeptide/oligopeptide
LC145144	ABC	3.A.1	dipeptide/oligopeptide
LC145145	ABC	3.A.1	dipeptide/oligopeptide
LC145135	FeoB	9.A.8	ferrous ion
LC145134	ABC	3.A.1	glycine betaine
LC145139	ABC	3.A.1	glycine betaine
LC145146	ABC	3.A.1	glycine betaine
LC145194	ABC	3.A.1	glycine betaine
LC145195	ABC	3.A.1	glycine betaine
LC145097	ABC	3.A.1	heme export
LC145120	ABC	3.A.1	heme export
LC145187	HCC	9.A.40	hemolysin C (HlyC) homolog
LC145188	MgtE	9.A.19	hemolysin C (HlyC) homolog
LC145109	ILT	9.A.10	iron ion
LC145142	ABC	3.A.1	lipid A
LC145104	ABC	3.A.1	lipoprotein
LC145122	ABC	3.A.1	lipoprotein
LC145176	ABC	3.A.1	lipoprotein
LC145208	ABC	3.A.1	lipoprotein
LC145212	ABC	3.A.1	lipoprotein
LC145105	ABC	3.A.1	lipoprotein releasing
LC145165	ABC	3.A.1	lipoprotein releasing
LC145207	ABC	3.A.1	lipoprotein releasing
LC145129	MgtE	9.A.19	magnesium ion
LC145111	MIT	1.A.35	magnesium/cobalt ion
LC145211	MIT	1.A.35	magnesium/cobalt ion
LC145115	ABC	3.A.1	manganese/zinc ion
LC145116	ABC	3.A.1	manganese/zinc ion
LC145128	ABC	3.A.1	manganese/zinc ion
LC145154	ABC	3.A.1	manganese/zinc ion
LC145099	ABC	3.A.1	mobybdenate
LC145100	ABC	3.A.1	molybdate
LC145149	ABC	3.A.1	multidrug
LC145106	MFS	2.A.1	multidrug efflux

LC145156	MOP	2.A.66	multidrug efflux
LC145163	MFS	2.A.1	multidrug efflux
LC145168	MOP	2.A.66	multidrug efflux
LC145190	MFS	2.A.1	multidrug efflux
LC145164	MFS	2.A.1	multidrug efflux (EmrB/QacA subfamily)
LC145192	MFS	2.A.1	multidrug efflux (EmrB/QacA subfamily)
LC145198	MFS	2.A.1	multidrug efflux (EmrB/QacA subfamily)
LC145130	RND	2.A.6	multidrug/solvent efflux (HAE1 subfamily)
LC145140	RND	2.A.6	multidrug/solvent efflux (HAE1 subfamily)
LC145189	RND	2.A.6	multidrug/solvent efflux (HAE1 subfamily)
LC145202	MFS	2.A.1	nitrate/nitrite
LC145213	ABC	3.A.1	oligopeptide
LC145096	ABC	3.A.1	phosphate
LC145173	ABC	3.A.1	phosphate
LC145174	ABC	3.A.1	phosphate
LC145175	ABC	3.A.1	phosphate
LC145185	ABC	3.A.1	phosphate
LC145121	ABC	3.A.1	phosphonates
LC145101	ABC	3.A.1	polyamine
LC145095	VIC	1.A.1	potassium ion channel
LC145119	CPA2	2.A.37	potassium/sodium ion:proton antiporter
LC145124	CPA2	2.A.37	potassium/sodium ion:proton antiporter
LC145110	Tat	2.A.64	protein export
LC145152	Tat	2.A.64	protein export
LC145125	F-ATPase	3.A.2	protons
LC145126	F-ATPase	3.A.2	protons
LC145151	F-ATPase	3.A.2	protons
LC145160	F-ATPase	3.A.2	protons
LC145161	F-ATPase	3.A.2	protons
LC145170	F-ATPase	3.A.2	protons
LC145204	F-ATPase	3.A.2	protons
LC145167	SBT	2.A.83	sodium ion:bicarbonate symporter
LC145193	CaCA	2.A.19	sodium ion:calcium ion antiporter
LC145107	SSS	2.A.21	sodium ion:proline symporter
LC145133	SSS	2.A.21	sodium ion:proline symporter
LC145136	SSS	2.A.21	sodium ion:proline symporter
LC145143	SSS	2.A.21	sodium ion:proline symporter
LC145166	SSS	2.A.21	sodium ion:proline symporter
LC145183	SSS	2.A.21	sodium ion:proline symporter
LC145141	ABC	3.A.1	toluene tolerance
LC145182	ABC	3.A.1	toluene tolerance
LC145094	IVSP	3.A.7	unknown substrate
LC145102	OOP	1.B.6	unknown substrate
LC145103	Mot/Exb	1.A.30	unknown substrate
LC145112	MTB	3.A.15	unknown substrate
LC145113	MTB	3.A.15	unknown substrate
LC145114	MTB	3.A.15	unknown substrate
LC145118	MTB	3.A.15	unknown substrate
LC145127	MTB	3.A.15	unknown substrate
LC145131	MTB	3.A.15	unknown substrate
LC145137	TRIC	1.A.62	unknown substrate
LC145138	Mot/Exb	1.A.30	unknown substrate
LC145155	DNA-T	3.A.11	unknown substrate
LC145157	IIISP	3.A.6	unknown substrate
LC145158	IIISP	3.A.6	unknown substrate
LC145159	IIISP	3.A.6	unknown substrate
LC145169	MTB	3.A.15	unknown substrate

LC145172	IIISP	3.A.6	unknown substrate
LC145177	IIISP	3.A.6	unknown substrate
LC145179	ABC	3.A.1	unknown substrate
LC145191	LIC	1.A.9	unknown substrate
LC145197	TRIC	1.A.62	unknown substrate
LC145201	IIISP	3.A.6	unknown substrate
LC145203	AEC	2.A.69	unknown substrate
LC145210	IIISP	3.A.6	unknown substrate
LC145147	MOP	2.A.66	virulence factor MviN
LC145196	ABC	3.A.1	zinc
LC145098	P-ATPase	3.A.3	zinc/cadmium/cobalt ion
LC145214	P-ATPase	3.A.3	zinc/cadmium/cobalt ion

Table S2. Primer sets used in this study.

Gene	Name (Accession no.)	Forward primer (5'-3')	Reverse primer (5'-3')	Product size (bp)
Sulfur-oxidation				
Sulfide dehydrogenase	*dhsU1* (AB735049)	CACACCAAAAGGCTGGAG	TTGGCATAGGTGTTGCAC	144
	dhsU2 (AB735050)	GTGTATCGGCAGCAAGACAA	GGAAAACAAAGGTTGCCAAA	142
Sulfide-quinone reductase	*sqrX* (AB735051)	TACCACCATTTGCAGGACAA	GCCAATCTGGACCATCAAG	144
	sqrF (AB735052)	TTGATAGTTGCGTTGGGAC	GGCTTTTGGATTACCACCA	168
Sulfite dehydrogenase	*sorA* (AB735181)	AACCTCTCCCAGCGGATAAC	CCCTCTTTGTTCCCAAAACC	134
Sulfur oxidation (sox) complex	*soxX* (AB735053)	GGGATAGAAAGTCCGGAAGG	TCAGGAGGAACGGCATAAAG	80
	soxY (AB735054)	GTATCTGCAGCCGAATTGG	AGCCTTTCCACCCGTTATC	84
	soxZ (AB735055)	AAGGACAAAGAAACGGGAA	CCGGATAAATCCATCCATG	98
	soxA (AB735056)	ATCCTGGGGAAGTTTTTGC	TCACTGTGGCAAGAAGCAC	94
	soxB (AB735057)	ACGGCATTAAGCCAAATACG	TAGGCAACACCACCCATC	97
Thiosulfate sulfurtransferase	*rhd1* (LC021536)	AAAGAAAAAGAGGCGATGCTC	TTGCTTGAAGGCTTCTATTTCC	118
	rhd2 (LC021537)	GCATTGGCTTTACAGGATG	GCTGTTTGGGGACATAGAGG	81
	rhd3 (LC021538)	CACCCCAAGAATACGAAAAAG	GCCAGAACACTTCTGTTTCCA	148
Respiration				
Cbb3 -type cytochrome *c* oxidase subunit I	*ccoN* (AB735065)	ATCGGCTTACAAGCAGCAG	TCGATCCACCAAGGAGTTTC	98
Carbon fixation (rTCA cycle)				
ATP citrate lyase subunit alpha	*aclA* (AB735068)	AACTTGGGCATCTGGATTTG	TTCCAGCTCTTGCGGTAACT	104
Fumarate hydratase	*fumA* (AB735071)	CCTTGGATTTGGAGGAACAA	CGGCAACTGGTAAAGATGC	83
2-oxoglutarate ferredoxin oxidoreductase subunit alpha	*forA* (AB735076)	TGTTACATGCGGAAAACCAA	TGCTACCGCAACAGGTATG	98
House keeping				
RNA polymerase, omega subunit	*rpoZ* (LC143896)	ACGAAACTGGTGCTGAGACA	TTTTTGCTTTCCCGGTTG	98
